# Accuracy of healthcare systems data for identifying cardiovascular outcomes after stroke due to intracerebral haemorrhage in the United Kingdom

**DOI:** 10.1186/s13063-024-08631-7

**Published:** 2024-11-16

**Authors:** Alice Hosking, Jacqueline Stephen, Jonathan Drever, William N. Whiteley, Cathie L. M. Sudlow, Rustam Al-Shahi Salman, for the RESTART Trial Steering Committee, William N. Whiteley, Cathie L. M. Sudlow, Rustam Al-Shahi Salman, Colin Baigent, Daniel Lasserson, Frank Sullivan, Johanna Carrie, Martin S Dennis, Gordon D Murray, David E Newby, Peter AG Sandercock, Nikola Sprigg, David J Werring, Phil M White

**Affiliations:** 1 Centre for Clinical Brain Sciences, The University of Edinburgh, Edinburgh, UK; 2 The Usher Institute, The University of Edinburgh, Edinburgh, UK; 3 Edinburgh Clinical Trials Unit, The Usher Institute, The University of Edinburgh, Edinburgh, UK; 4 British Heart Foundation Data Science Centre, Health Data Research UK, London, UK

**Keywords:** Healthcare systems data; Intracerebral haemorrhage; Trial outcome adjudication

## Abstract

**Background:**

Healthcare systems data (HCSD) could improve the efficiency of clinical trials, but their accuracy and validity are uncertain. Our objective was to assess the accuracy of HCSD as the sole method of outcome detection in the REstart or STop Antithrombotics Randomised Trial (RESTART; ISRCTN71907627) compared with adjudicated questionnaire follow-up and compare estimates of treatment effect.

**Methods:**

RESTART was a prospective, open, assessor-blind, parallel-group randomised controlled trial (RCT) of antiplatelet therapy after intracerebral haemorrhage (ICH) in the UK.

We included 496 (92%) of 537 RESTART participants, who were resident in England or Scotland at randomisation. Computerised randomisation incorporating minimisation allocated participants (1:1) to start or avoid antiplatelet therapy.

RESTART used annual questionnaires to detect its primary outcome (recurrent ICH) and secondary outcome (a composite of haemorrhagic or ischemic major adverse cardiovascular events [MACE]) over a median of 2.0 years; an independent adjudication committee verified outcomes using medical records and brain imaging. We obtained ICD10-coded HCSD on hospital admissions and deaths in England and Scotland to identify primary and secondary outcomes. We compared HCSD with a reference standard of adjudicated outcomes. We estimated the effects of antiplatelet therapy using HCSD alone in a Cox proportional hazards model adjusted for minimisation variables.

**Results:**

In the original RESTART trial, 31 people experienced a primary outcome event. HCSD had sensitivity of 84% (95% CI 66 to 95%) and positive predictive value of 68% (51 to 82%) for recurrent ICH. HCSD estimated an effect of antiplatelet therapy (adjusted hazard ratio [aHR] 0.51, 95% CI 0.27 to 0.98; *p* = 0.044) that was almost identical to adjudicated outcomes (aHR 0.51, 95% CI 0.25 to 1.03; *p* = 0.060). HCSD had sensitivity of 84% (76 to 91%) and positive predictive value of 78% (69 to 85%) for MACE, on which HCSD estimated an effect of antiplatelet therapy (aHR 0.81, 95% CI 0.56 to 1.16; *p* = 0.247) that was similar to adjudicated outcomes (aHR 0.65, 95% CI 0.44 to 0.95; *p* = 0.025).

**Conclusions:**

In a RCT of antiplatelet therapy for people with ICH, HCSD was reasonably accurate and provided similar estimates of treatment effect compared with adjudicated outcomes.

**Trial registration:**

ISRCTN71907627. Registered on 25 April 2013.

## Background

Over the last half-century, carefully conducted, robust randomised controlled trials (RCTs) have informed the secondary prevention of cardiovascular diseases in everyday clinical practice. In stroke medicine, RCTs have shown the benefits of antihypertensive therapy [1], statin therapy [2], carotid endarterectomy [3], antiplatelet therapy [4], and dual antiplatelet therapy [5]. For intracerebral haemorrhage (ICH), RCTs have provided evidence of the benefits of acute [6] and long-term blood pressure management [7], and intensive care bundles [8]. However, RCTs are expensive, time-consuming, and carbon-intensive to conduct because of their staffing and resource requirements. These resource requirements include the burden of face-to-face, postal, or remote methods of follow-up for outcomes and their adjudication, which also squeeze RCT budgets and limit the duration of follow-up [9, 10].

Healthcare systems data (HCSD) from electronic health records, administrative databases, or disease registers can be used to design and streamline participant identification, recruitment, consent, randomisation, follow-up, and detect clinical outcome events in RCTs. HCSD has the potential to improve the convenience and reduce the cost of RCTs for cardiovascular disease and for stroke specifically [11, 12]. The proportion of RCTs in the UK using HCSD for detection of primary or secondary outcomes appears to be increasing since 2019 [13]. To be confident in RCTs’ results, outcome data need to be as accurate, complete, and unbiased as possible, resulting in valid and reliable estimates of treatments’ effects. For RCTs using HCSD to identify outcomes, utility assessments comparing HCSD to RCTs’ alternative primary methods of outcome detection should inform whether HCSD meet these requirements, but few such assessments exist in the UK or elsewhere [14, 15]. A systematic review of ten RCTs of cardiovascular event prevention found good agreement between HCSD and adjudicated outcomes for death and some cardiovascular outcomes, with similar directions and magnitudes of treatment effects [16]. In individual RCTs comparing HCSD with adjudicated outcomes, one RCT of aspirin for primary prevention of cardiovascular events had similar findings including very similar effects of aspirin on major bleeding [17]. However, the completeness of HCSD was insufficient for detecting cardiovascular outcomes in a prostate cancer RCT [18].

There is uncertainty about the utility of HCSD for ascertaining cardiovascular outcomes and determining treatment effects in people with stroke and especially people with multiple cardiovascular diseases. This population is particularly challenging because of the occurrence of both ischemic and haemorrhagic major adverse cardiovascular events (MACE). These can be difficult to distinguish from non-specific International Classification of Diseases 10th Revision (ICD-10) codes, can recur within the same hospital admission, can result in multiple hospital admissions for the same event, or can be attributed as the primary cause of death in someone who dies from the consequences of disability long after a MACE.

Therefore, we sought to compare the accuracy and treatment effect estimates of HCSD (as the sole method of follow-up) versus adjudicated cardiovascular outcomes detected by questionnaires in a prospectively planned study within the Restart or Stop Antithrombotics Randomised Trial (RESTART; ISRCTN71907627) of long-term antiplatelet therapy after stroke due to intracerebral haemorrhage (ICH), which was conducted in the UK between 2013 and 2018 [19].

## Methods

### Study design and participants

RESTART was a prospective, open, assessor-blinded, parallel-group RCT that included 537 participants at 122 hospitals in the UK (England, Scotland, Wales and Northern Ireland) between May 22, 2013, through May 31, 2018. Participants were aged ≥ 18 years and had taken antithrombotic therapy for the prevention of occlusive vascular disease when they developed spontaneous (non-traumatic) symptomatic ICH, discontinued antithrombotic therapy, and survived for 24 h. Participants were randomised to either start or avoid antiplatelet therapy. The protocol pre-specified a primary outcome of recurrent symptomatic ICH [20, 21]. The protocol also pre-specified a composite secondary outcome of all MACE defined by the Antithrombotic Trialists’ Collaboration (nonfatal myocardial infarction, nonfatal stroke [ischemic, haemorrhagic, or uncertain cause], or death from a vascular/unknown cause) [22].

The funding application and trial protocol pre-specified that HCSD would be collected alongside other methods to identify outcome events, for which participants gave consent.

### Test methods

In RESTART, outcome events were ascertained using structured postal or telephone questionnaires completed annually by each participant (or their representative) and their primary care doctor, and from spontaneous reporting by trial sites. All outcome events underwent independent clinical adjudication by two consultant neurologists, who were blinded to treatment allocation and use of antithrombotic therapy, using all available source documentation from medical records and brain imaging. Participants’ follow-up lasted between 6 months and 5 years until November 30, 2018 (median 2.0 years, inter-quartile range [IQR] 1.0–3.0) in all participants bar one who withdrew before the first follow-up [19, 23].

In this study, the index test for outcome events was HCSD. After trial recruitment and follow-up were complete, we requested HCSD from administrative databases of ICD-10-coded hospital admissions and deaths in England (via NHS England’s Data Access Request Service) and Scotland (via Public Health Scotland’s electronic Data Research and Innovation Service), but not Northern Ireland (where they were not available for research) or Wales (due to administrative issues), thereby including the two largest UK nations where 92% of the RESTART cohort resided when they were recruited. English hospital admissions are recorded in Hospital Episode Statistics – Admitted Patient Care (HES-APC), with up to twenty diagnoses recorded as ICD-10 codes for each admission by administrators based on medical documentation at hospital discharge. Scottish hospital admissions are recorded in Scottish Morbidity Records (SMR01), with a ‘main condition’ and up to five ‘other conditions’ recorded as ICD-10 codes for each admission by administrators based on medical documentation at hospital discharge. Medical certificates of causes of death in England and Scotland involve doctors recording the disease or condition leading directly to death (part 1a), other diseases or conditions leading to 1a including the underlying cause of death, and other significant conditions contributing to death but not related to the disease or condition causing (part 2); these records are coded using ICD-10 and held by NHS Digital in England and National Records of Scotland.

We requested data between each participant’s randomisation date and November 30, 2018. We mapped recurrent ICH and components of the composite outcome of all MACE (defined by the Antithrombotic Trialists’ Collaboration) to ICD-10 codes (Table 1), with reference to the CALIBER chronological map of human health [24], the Antithrombotic Triallists [25] and 2002 meta-analyses [25, 26], UK Biobank definitions of stroke and myocardial infarction [27, 28], and record linkage studies of ICH [29, 30]. We searched for these pre-specified codes in fields for the disease or condition leading directly to death or the first/main diagnostic code for each hospital admission.

**Table 1:** ICD-10 codes used to identify outcomes in healthcare systems data

Outcome	ICD-10 code(s) used to identify outcomes in HCSD
*Primary outcome*	
Intracerebral haemorrhage	I61.x
*Secondary outcome*	
* MACE composite of all of the following:*	
Myocardial infarction (ST elevation or non-ST-elevation)	I21.x, I22.x
Ischemic stroke	I63.x
Symptomatic stroke of uncertain sub-type	I63.9, I64.x, G46.x
Symptomatic recurrent intracerebral haemorrhage^a^	I61.x
Symptomatic spontaneous intracranial haemorrhage	I60.x, I62.x, I69.0, I69.2
Symptomatic major extracranial haemorrhage^a^	N83.7, O71.7, O90.2, T81.0, H35.6, H43.1, H45.0, K92.0, K92.1, K92.2
Mesenteric ischemia^a^	K55.02, K55.04, K55.06
Symptomatic peripheral arterial occlusion^a^	I73.1, I73.8, I73.9, I74.3, I74.4, I74.5
Symptomatic deep vein thrombosis^a^	I82.4, I82.6
Symptomatic pulmonary embolism^a^	I26.x
Sudden cardiac death	I47.2, I49.0, I46.0, I46.9, I47.0
Other cardiovascular death	I20.x to I25.x, I30.x to I52.x, I60.x to I69.x, I70.x to I79.x, I80.x to I89.x
Deaths of undetermined cause	R99

*HCSD*, healthcare systems data; *ICD-10*, 10th version of the International Classification of Diseases

I61.x means I61 and all subsidiary codes

^a^Only fatal events were included in the composite MACE outcome to adhere to the Antithrombotic Trialists’ collaboration definition

In this study, the reference standard was the best-available outcome data. Any hospital admissions or deaths detected by HCSD that did not match an outcome event or serious adverse event known to RESTART to within ± 1 day were independently adjudicated with source documentation to identify any outcome events that were missed by RESTART. This information was combined with the RESTART adjudicated outcomes to give the reference standard.

### Analysis

We did not perform a sample size calculation but used the largest dataset available to us by including all RESTART participants who had consented to data linkage and were resident in England or Scotland when randomised. We restricted analyses to the first recurrent ICH or MACE outcome event during follow-up, using ICD-10 codes in the primary diagnostic position in HCSD.

#### Accuracy of HCSD

We restricted analyses to hospital admissions and deaths following the date a participant was discharged from hospital after randomisation in RESTART (to avoid identifying the qualifying ICH as an outcome event) and before the date of their last questionnaire follow-up. Measures of diagnostic accuracy used the following definitions. True positives were outcome events identified by both the index test (HCSD) and the reference standard (adjudicated questionnaire follow-up and site reporting in RESTART combined with adjudicated HCSD). False positives were outcomes identified by the index test, but not by the reference standard. False negatives were outcomes identified by the reference standard but not by the index test. True negatives (i.e. people without an outcome in either dataset) could not be identified. We calculated the sensitivity and positive predictive value (PPV) of HCSD with exact 95% confidence intervals (CI).

#### Treatment effect estimates using HCSD

We estimated the effect of starting versus avoiding antiplatelet therapy for the included participants according to the treatment strategy assigned at randomisation using outcome events identified by HCSD alone (without reference to RESTART’s adjudicated outcomes) until November 30, 2018 (allowing longer follow-up than the date of each participant’s last completed questionnaire). We estimated the survival in each treatment group using a Kaplan–Meier survival analysis of time to first occurrence of an outcome event during all available follow-up after randomisation, censored at death unrelated to an outcome event or last available follow-up. We assessed the proportional hazards assumption graphically and including a treatment by log(time) interaction, and if it was met, we constructed a Cox proportional hazards regression model adjusted for all five covariates included in the minimisation algorithm to calculate an adjusted hazard ratios (aHR) as was done in RESTART [19].

## Results

### Participants and outcomes

**Fig. 1:**
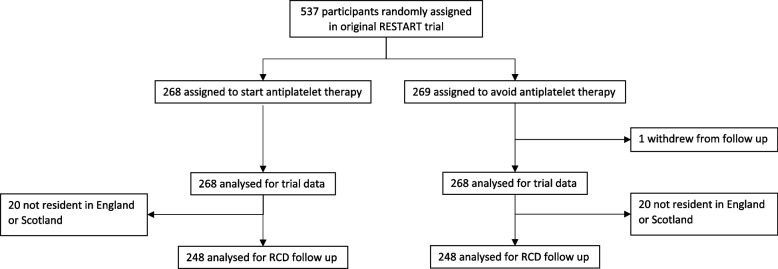
RESTART participants suitable for follow-up via healthcare systems data in England and Scotland

The baseline characteristics and prognosis of participants in RESTART have been reported elsewhere [19]. Of 537 RESTART participants, one withdrew from follow-up, 29 were resident in Wales and 11 in Northern Ireland, leaving 496 (92%) who were resident in England and Scotland at randomisation to be included in these analyses (Fig. 1).

### Accuracy of HCSD

Amongst the 496 participants, HCSD identified 64 recurrent ICH and 194 MACE events from ICD-10 codes, of which 51 and 168 respectively were in the primary diagnostic position, and 38 and 104 respectively were first events that occurred between hospital discharge following randomisation and before each participant’s last follow-up. RESTART identified 31 first instances of recurrent ICH during trial follow-up, whereas HCSD identified 38 events in the same period. There were 26 true positives, 5 false negatives, and 12 false positives, giving a sensitivity of 84% (95% CI 66 to 95%) and PPV of 68% (51 to 82%) (Table 2). HCSD did not identify new primary outcomes that had been missed by adjudicated questionnaires. RESTART identified 96 first instances of MACE during trial follow-up, whereas HCSD identified 104 events in the same period. There were 81 true positives, 15 false negatives, and 23 false positives, giving a sensitivity of 84% (95% CI 76 to 91%) and PPV of 78% (95% CI 69 to 85%) (Table 2). HCSD identified three new MACE outcomes that had been missed by adjudicated questionnaires.

**Table 2:** First outcomes during follow-up after randomisation in healthcare systems data compared to adjudicated outcomes

		**Healthcare systems data**			
		ICD-10 outcome code	No ICD-10 outcome code	Sensitivity (95% CI)	PPV (95% CI)
**Adjudicated outcomes detected using annual questionnaires**	*Recurrent ICH*				
	Outcome occurred	26^a^	5^b^	84% (66 to 95%)	68% (51 to 82%)
	Outcome did not occur	12^c^	x		
	*MACE*				
	Outcome occurred	81^d^	15^e^	84% (76 to 91%)	78% (69 to 85%)
	Outcome did not occur	23^f^	x		

*PPV*, positive predictive value

^a^1 admission matched the second recurrent ICH, but not the first (which occurred outside the UK)

^b^2 missed due to coding (I60.9 and I62.9), 2 had no recorded hospital admission, and 1 occurred during a hospital admission for another outcome

^c^8 hospital admissions/deaths were not due to recurrent ICH and 4 were MACE outcomes but not recurrent ICH

^d^3 admissions identified outcomes that were missed by RESTART but are included in the reference standard

^e^7 missed due to coding (I24.9, I60.9, I67.9, I25.1, I50.0, H53.8, and J18.9), 7 had no recorded hospital admission, and 1 occurred during a hospital admission for another outcome

^f^11 hospital admissions/deaths were not due to MACE and 12 were outcomes but not MACE

### Treatment effect estimates using HCSD

**Fig. 2:**
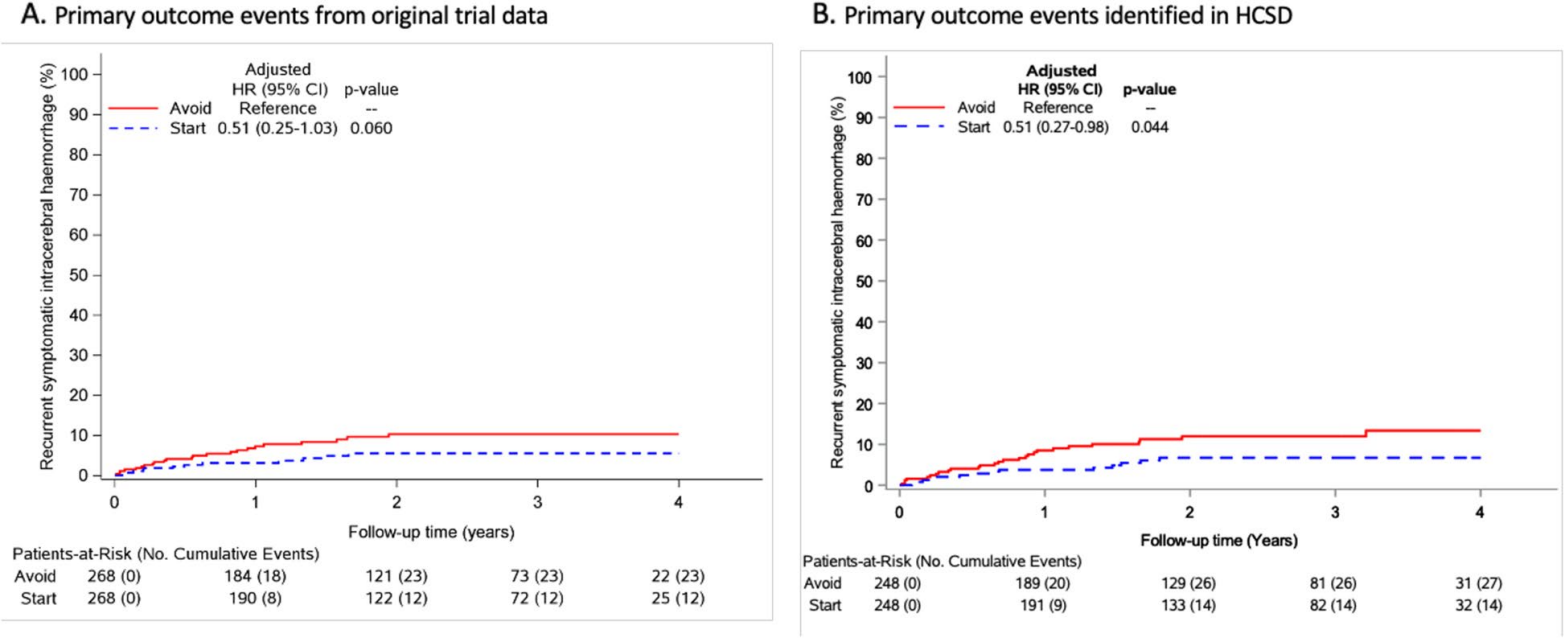
Comparison of the effects of antiplatelet therapy on RESTART’s primary outcome of recurrent ICH using adjudicated outcomes detected by annual questionnaires for 536 participants with follow-up in the UK (**A**) [19] or outcomes identified using healthcare systems data for 496 participants resident in England or Scotland (**B**)

**Fig. 3:**
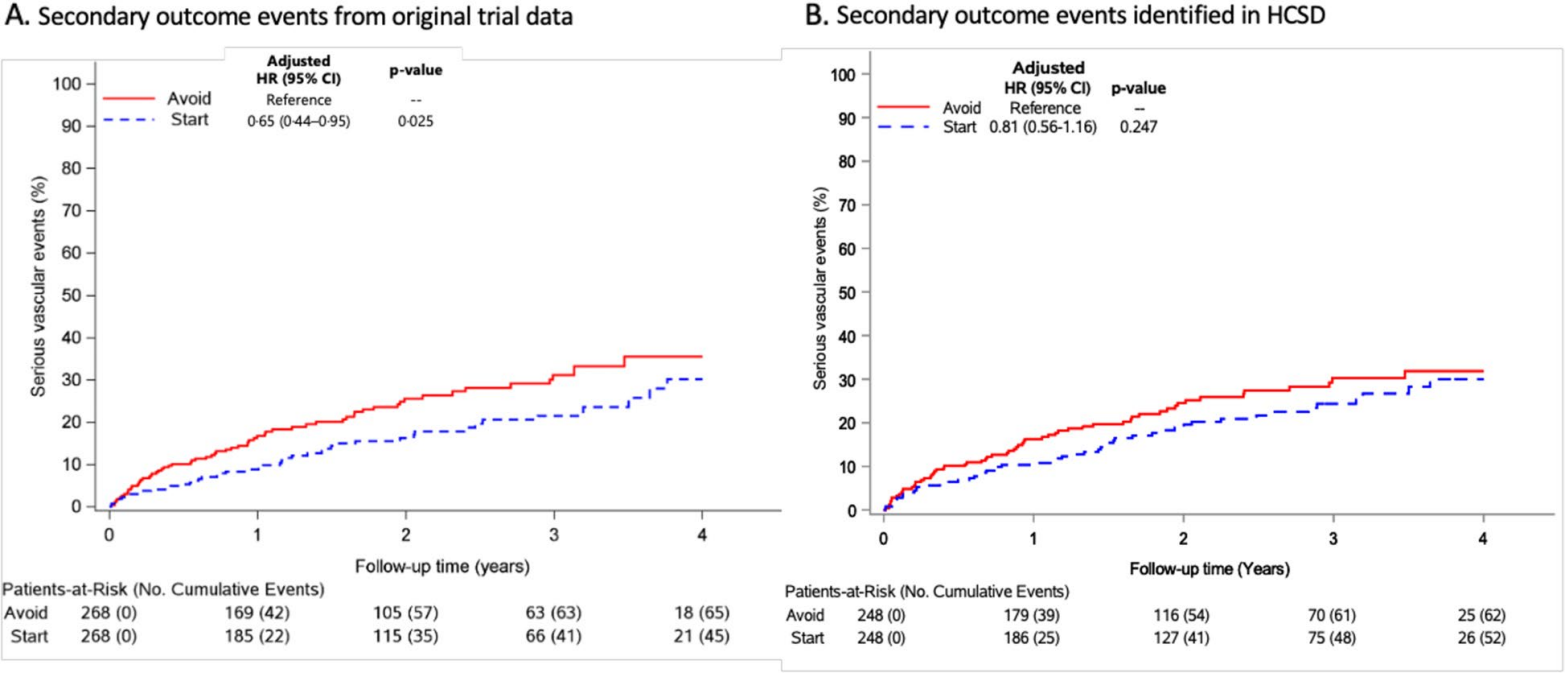
Comparison of the effects of antiplatelet therapy on RESTART’s secondary outcome of major adverse cardiovascular events (MACE, as defined by the Antithrombotic Trialists’ Collaboration [22] using adjudicated outcomes detected by annual questionnaires for 536 participants with follow-up in the UK (**A**) [19] or outcomes identified using healthcare systems data for 496 participants resident in England or Scotland (**B**)

Amongst the 496 participants, HCSD identified 41 recurrent ICH and 114 MACE events that occurred between hospital discharge following randomisation and the end of the trial on November 30, 2018. In RESTART, 35 first recurrent ICH and 110 MACE occurred during follow-up of 536 participants. The direction and magnitude of the effect of antiplatelet therapy on recurrent ICH was identical in RESTART (aHR 0.51, 95% CI 0.25–1.03; *p* = 0.060) and HCSD (HR = 0.51, 95% CI 0.27–0.98; *p* = 0.044), although HCSD was more precise and identified a statistically significant effect (Fig. 2). The direction and magnitude of the effect of antiplatelet therapy on MACE was similar in RESTART (aHR 0.65, 95% CI 0.44–0.95; *p* = 0.025) and HCSD (HR = 0.81, 95% CI 0.56–1.16; *p* = 0.247), although the magnitude of the point estimate of effect was somewhat attenuated in HCSD (Fig. 3).

## Discussion

In prospectively planned analyses of HCSD conducted after the results of RESTART were known, we found that HCSD had reasonable diagnostic accuracy when compared with adjudicated outcomes. HCSD alone gave an identical estimate of treatment effect on the primary outcome of recurrent ICH (which became statistically significant) and a similar estimate of treatment effect on the secondary outcome of MACE. This suggests that biases in the ascertainment of events were similar for the two methods.

Our study has strengths: we included participants from two UK nations in the only RCT of antiplatelet therapy after ICH. HCSD performed well despite the population being multimorbid with multiple cardiovascular diseases. Whereas previous studies in this area have compared HCSD to the trial outcome [31], we undertook a thorough process of outcome adjudication and were able to compare HCSD to a best-available outcome, to give a truer estimate of the sensitivity and specificity of HCSD.

The weaknesses of our study are that we were not able to analyse the HCSD for the full original trial population of our relatively small trial, due to administrative and governance issues, so we did not link data for the 41 people living in Northern Ireland and Wales. The study design is susceptible to incorporation bias, which occurs when the reference standard incorporates the test under study. Our reference standard included HCSD with the adjudicated trial outcome, but this did not apply to the primary outcome and only affected the secondary outcome (we included three MACE outcomes identified by HCSD in the reference standard). Analyses may have been affected by unquantified migration of patients between and from UK nations, which would lead to an underestimate of the accuracy of HCSD, but we expect migration rates to be low in relatively elderly population. There is no agreement about how accurate HCSD need to be for use in RCT follow-up, so our values for PPV and sensitivity must be interpreted in the context of similarities in treatment effects. Our reference standard is imperfect, because traditional trial outcome ascertainment with adjudication has limitations. It is reliant on reporting of events by site investigators; source documentation can be incomplete or inconsistent; and adjudicators often disagree, underlining the inherent subjectivity in the process [32].

Our results are relevant to MACE in a secondary prevention RCT after ICH. There may be condition-specific issues with HCSD which reduce the generalizability of our results. In observational studies, definitions used for MACE in HCSD vary, prohibiting comparison, aggregation, and replication of findings [33]. Further work is needed to comprehensively understand the accuracy of coding of cardiovascular disease outcomes in modern healthcare systems. Countries and regions have different systems and incentives for recording healthcare data which may bias results [34], and our results only apply to England and Scotland.

Previous studies have shown that the result of a clinical trial obtained through HCSD may be closer to null than when obtained through traditional follow-up methods, implying that larger sample sizes might be needed if planning follow-up with HCSD [16]. More recently, a comparison of major bleeding events recorded as adjudicated outcomes in the ASCEND trial compared to HCSD found no clinically important differences in the relative risk with treatment of events depending on data source used [35]. The result we obtained for RESTART using HCSD for the primary outcome had an identical hazard ratio to the original trial, and for secondary outcomes, the hazard ratio was similar. The accuracy of HCSD is limited by correct diagnostic coding in the data. It may be that improvements in electronic healthcare records over the past decade have increased the accuracy of HCSD. The accuracy of recording of outcome events in HCSD for a study of bladder cancer in England improved substantially between 2011 and 2017, with remuneration policies likely driving the improvement in data quality [36]. Many of the studies of HCSD in cardiovascular disease were conducted years ago, such as the West of Scotland Coronary Prevention Study, which originally began recruiting in 1995 [31].

In the current study, HCSD identified both false positive and false negative events, compared to the best-available data. The relatively large number of false positives reduced the PPV of HCSD. Our experience suggests that re-admissions can often attract incorrect coding, leading to false positives. Future work could focus on how to identify these events without sacrificing completeness. Two primary outcome events and seven secondary outcome events were false negatives due to miscoding. Access to alternative sources of information, such as national audit data or primary care data, would allow triangulation of outcomes and potentially increase the accuracy of HCSD.

Our sample size was too small to explore whether HCSD missed mild events that did not result in hospital admission or death, but were detected by RESTART. These events could be detected with access to primary care records, but linking RCT data to primary care records is currently not possible in England due to data governance and data ownership constraints [37]. Widening the scope of access could improve the accuracy of HCSD for all clinical research.

HCSD is being used in RCTs to enhance data collection, as in the RECOVERY platform trial in COVID-19 [38], for very long-term follow-up [39], and as exclusive outcome follow-up [40, 41]. Although digitally enabled RCTs seem to offer benefits, researchers in the BladderPath RCT of bladder cancer treatment pathways found rapid access to outcomes from digital sources was ‘too cumbersome and expensive’ in England for incorporation into the RCT design. The same authors were able to use HCSD for one-off long-term follow-up of another RCT [42]. The MRC Clinical Trials Unit has described the protracted process of applications for HCSD from NHS Digital and the National Institute for Cardiovascular Outcomes Research, which in the case of the PATCH trial took several years [43].

In this study, we have demonstrated that HCSD has good accuracy for identifying cardiovascular outcome events and effect estimates that are comparable to adjudicated outcome events. A Delphi consensus has prioritised the remaining challenges for use of HCSD in RCTs [44]. Further work is needed to confirm the accuracy of HCSD for cardiovascular research in other healthcare systems and to enable other types of informative HCSD to be efficiently incorporated into RCTs.

## Conclusions

In a RCT of antiplatelet therapy for people with ICH, HCSD was reasonably accurate and provided similar estimates of treatment effect compared with adjudicated outcomes. Triallists could consider using healthcare systems data to detect major adverse cardiovascular events after intracerebral haemorrhage

## Data Availability

A fully anonymised version of the original RESTART trial dataset used for analysis with individual participant data and a data dictionary is available for researchers to apply to use, via https://datashare.is.ed.ac.uk/handle/10283/3265. Written proposals will be assessed by members of the RESTART trial steering committee and a decision made about the appropriateness of the use of data. Healthcare systems data are stored in national safe havens and were accessed for one-off use and so are not available for sharing.

## Abbreviations

aHR: Adjusted hazard ratio
HCSD: Healthcare systems data
ICD-10: International Classification of Diseases 10th Revision
ICH: Intracerebral haemorrhage
MACE: Major adverse cardiovascular events
MRC: Medical Research Council
PPV: Positive predictive value
RCT: Randomised controlled trial
RESTART: REstart or STop Antithrombotics Randomised Trial
